# Maternal Supplementation of Probiotics, Prebiotics or Postbiotics to Prevent Offspring Metabolic Syndrome: The Gap between Preclinical Results and Clinical Translation

**DOI:** 10.3390/ijms231710173

**Published:** 2022-09-05

**Authors:** Ying-Hua Huang, You-Lin Tain, Chien-Ning Hsu

**Affiliations:** 1 Department of Pediatrics, Kaohsiung Chang Gung Memorial Hospital, Kaohsiung 833, Taiwan; 2 College of Medicine, Chang Gung University, Taoyuan 333, Taiwan; 3 Department of Pharmacy, Kaohsiung Chang Gung Memorial Hospital, Kaohsiung 833, Taiwan; 4 School of Pharmacy, Kaohsiung Medical University, Kaohsiung 807, Taiwan

**Keywords:** obesity, hypertension, metabolic syndrome, hyperlipidemia, probiotics, prebiotics, postbiotics, developmental origins of health and disease (DOHaD)

## Abstract

Metabolic syndrome (MetS) is an extremely prevalent complex trait and it can originate in early life. This concept is now being termed the developmental origins of health and disease (DOHaD). Increasing evidence supports that disturbance of gut microbiota influences various risk factors of MetS. The DOHaD theory provides an innovative strategy to prevent MetS through early intervention (i.e., reprogramming). In this review, we summarize the existing literature that supports how environmental cues induced MetS of developmental origins and the interplay between gut microbiota and other fundamental underlying mechanisms. We also present an overview of experimental animal models addressing implementation of gut microbiota-targeted reprogramming interventions to avert the programming of MetS. Even with growing evidence from animal studies supporting the uses of gut microbiota-targeted therapies start before birth to protect against MetS of developmental origins, their effects on pregnant women are still unknown and these results require further clinical translation.

## 1. Introduction

Metabolic syndrome (MetS) is a group of concurrent medical conditions that raise risk of cardiovascular disease (CVD). The major components of MetS are obesity, hypertension, dyslipidemia and insulin resistance [1]. It is estimated that around one-quarter of the world population (one billion) is affected by MetS [2]. Of note is that MetS and associated disorders constitute two thirds of the non-communicable diseases (NCDs), the leading causes of death globally [3]. Without specific therapeutic regimens for diverse phenotypes of MetS, its prevalence is rising worldwide [2]. Hence, a strategic approach to avert the spread of MetS should be switched from disease treatment to prevention.

Recent epidemiological and experimental studies suggest that metabolic syndrome can originate in early life [4,5,6,7]. Exposure to various environmental cues in early life can alter organ structure and function that may raise the risk for developing MetS in later life [4,5,6,7]. This notion is now being termed the developmental origins of health and disease (DOHaD) [8].

Notably, different environmental insults in early life can program similar features of MetS, proposing a commonality of mechanistic pathways behind MetS of developmental origins. Despite these pathogenic mechanisms underlying developmental programming are still inconclusive, several common mechanisms have been reported, including nitric oxide (NO) deficiency, oxidative stress, aberrant activation of the renin-angiotensin-aldosterone system (RAAS), dysfunctional nutrient sensing signals, epigenetic regulation and gut microbiota dysbiosis [4,5,6,7,9,10,11,12].

Recent research has highlighted the influence of the gut microbiota in MetS and associated disorders [13]. Gut microbiota derived metabolites can work as signaling compounds through systemic circulation involving in human disease, including MetS [13]. Fetal exposure to environmental insults has been connected with negative impact on offspring gut microbiota maturation, which precede later onset of disease in adult life [14]. Nevertheless, relatively little information exists regarding whether and how various maternal insults could shape gut microbiota, leading to MetS and associated disorders in adult offspring.

Conversely, unfavorable programming processes can be averted by intervention in early life to stop or delay the development of chronic diseases throughout life, which is referred to as reprogramming [15]. Considering gut microbiota dysbiosis is closely connected with the developmental of MetS, interventions gut microbiota and relevant metabolites may serve as a potential target for therapeutics [16,17,18,19].

Probiotics (i.e., beneficial microorganisms), prebiotics (i.e., compounds in food can assist the growth of probiotics) and postbiotics (i.e., metabolites of probiotics providing physiological benefits) are commonly used gut microbiota-targeted therapies. Our review aims to map the fundamental concepts in how the uses of probiotics, prebiotics and postbiotics in early life prevent the developmental programming of MetS.

We searched the MEDLINE/PubMed and Embase databases for studies written in English between January 1980 and July 2022 using the following list of keywords: “gut microbiota”, “probiotics”, “prebiotics”, “synbiotics”, “postbiotics”, “parabiotics”, “cardiovascular disease”, “cardiometabolic disorder”, “developmental programming”, “DOHaD”, “reprogramming”, “dyslipidemia”, “hyperlipidemia”, “obesity”, “diabetes”, “insulin resistance”, “hyperglycemia”, “hypertension”, “mother”, “father”, “gestation”, “pregnancy”, “progeny”, “offspring” and “metabolic syndrome”. Additional studies were selected and evaluated based on references in eligible literature. The search was ended by 10 July 2022.

## 2. Current Evidence Supports MetS of Developmental Origins

### 2.1. Epidemiological Evidence

There is tremendously epidemiological evidence suggesting that negative early-life conditions are associated with the risk of MetS later in life. First, available data indicate that famines increase risk of developing MetS [20,21,22,23]. The Dutch Famine Birth Cohort Study revealed that pregnant women under famine had children who developed obesity, hypertension, dyslipidemia and insulin resistance [20,22]. Studies in other famines show similar effects [21,23]. Another line of evidence supports MetS of developmental origins coming from many observational studies of risk factors. Risk factors reported in these studies relating to MetS and associated disorders include environmental chemicals exposure [24], maternal obesity [25,26], gestational diabetes [26,27] and excessive postnatal weight gain [28]. Third, data from twin pregnancy revealed that there was an association between low birth weight (LBW) and certain features of MetS [29,30]. Lastly, a systematic review recruiting 39 studies demonstrated that rapid weight gain in infant with LBW had an around 80% great risk for CVDs [31]. From these observations, there might be a relationship between early-life environmental exposure, fetal programming and the development of MetS later in life.

Notably, these observational studies are not able to offer molecular mechanisms underlying programming processes of MetS for the creation of reprogramming interventions. Accordingly, the biological plausibility of the associations, proof of causality and development of reprogramming strategies have long been reliant on evidence whereby animal models stand.

### 2.2. Experimental Evidence

Considering the difficulties in building animal models that exhibit all the components of MetS, studying MetS of developmental origins are performed using models that manifest certain, but not all, characteristics of MetS in most investigations [4,5,6,7]. According to the experimental approach, several species such as rats [32], mice [33], rabbits [34], sheep [35], pigs [36] and non-human primate [37] have been used to evaluate developmental programming of MetS. Among them, rats are most commonly used animals for comparisons of major features of MetS develop throughout the lifetime [7]. Several environmental insults in early life have been reported to program certain features of MetS in adult offspring, containing maternal imbalanced nutrition, maternal illness, environmental chemical exposure, medication use, etc. [4,5,6,7,27,28,29,30].

#### 2.2.1. Maternal Nutrition Imbalance

The array of maternal imbalanced nutrition that have been established to induce different features of MetS is categorized into models that aim to restrict calorie intake, restrict certain nutrients or increase consumption of specific nutrients. Several maternal nutrient restriction models have been created to mimic the malnutrition experienced by pregnant women exposed to famine.

Caloric restriction is a dietary regimen that reduces energy intake without incurring specific nutrient. Restriction of calories ranging from 30% to 70% in dams has been stated to cause offspring hypertension, a key characteristic of MetS [38]. In addition to hypertension, severe 70% maternal caloric restriction resulted in obesity, hyperleptinemia and insulin resistance in adult offspring [39]. The severity of deleterious consequence seems related to the degree of caloric restriction and the timing of exposure [38,40].

The protein restriction model is the same as the caloric restriction model that mimics the challenge faced in developing countries. In rats, protein restriction with a range from 6–9% to pregnant dams resulted in offspring hypertension [38]. Rodent studies of maternal protein restriction also result in intrauterine growth retardation (IUGR) with subsequent insulin resistance, obesity, hyperglycemia, glucose intolerance and adipocyte hypertrophy [41].

There is also evidence to endorse that deficiencies in certain nutrients in pregnant mothers resulting in MetS in adult progeny. In rodent models, when deficiencies in iron [42], zinc [43], sodium [44], calcium [45], vitamin D [46] or methyl donor nutrients (folic acid; methionine; choline; vitamins B2, B6 and B12) [47], in dams, their adult offspring were likely to have elevated BP [38]. In addition, offspring of pregnant mothers with low levels of trace elements and vitamins are at risk for developing MetS-related phenotypes, such as insulin resistance [48,49], impaired glucose tolerance [50], increased visceral adiposity and altered lipid metabolism [51].

On the other hand, the excessive consumption of specific nutrients can also program MetS and associated disorders in adult offspring [25]. The Western diet is characterized for being rich in saturated fats, salt and refined sugars. Animal models of maternal diets containing key components of the human Western diet, synergistic effects of fat, salt and refined sugars on the elevation of BP in adult offspring were noticed [52,53,54]. Rodent models of high-fat diet-induced obesity have been used widely to study human obesity-related disorders [55,56]. Numerous animal studies reveal that maternal high-fat diet can program MetS traits in adult rat progeny, such as hypertension [57], obesity [58], dyslipidemia [59] and insulin resistance [59].

Much of the increase in sugar consumption is from high-fructose corn syrup and refined sugars [60]. Prior work indicates that intake of high-fructose alone or as a part of diet by rodent mothers induces multiple characteristics of MetS in adult progeny, such as hypertension, obesity, insulin resistance, dyslipidemia and hepatic steatosis [61,62,63].

#### 2.2.2. Maternal Illness

Maternal illness and/or complications during pregnancy impact fetal programming, which can be marked by IUGR [64]. IUGR offspring displayed dyslipidemia, hypertension and insulin resistance in a rat model of uteroplacental insufficiency [65,66]. So far, several animal models have been built resembling various maternal illnesses to evaluate MetS of developmental origins, including polycystic ovary syndrome (PCOS) [67,68], hypoxia [69], inflammation [70,71], diabetes [72,73,74] and chronodisruption [75,76].

In the PCOS model, adult offspring manifested dyslipidemia and hypertension at 16–17 weeks of age [67,68]. Maternal hypoxia and inflammation are also able to induce MetS-related phenotypes in adult rat progeny, including hypertension [69,70], obesity [69] and insulin resistance [71]. Additionally, rodent studies of maternal diabetes induced by streptozotocin (STZ) cause various features of MetS in offspring, such as insulin resistance, obesity, dyslipidemia, hypertension and CVDs [72,73,74].

Since the circadian system is the principal regulator of metabolism, circadian rhythm sleep disorders have been linked to MetS [77]. Data from animal studies indicated that maternal constant light exposure or dams received pinealectomy can program offspring’s hypertension [75] and insulin resistance [76].

#### 2.2.3. Exposures to Chemicals or Drugs

Prior review showed adult rats exposed to several chemicals during early life developed hypertension, a major feature of MetS [78]. These chemicals, while only bisphenol A and di-(2-ethylhexyl) phthalate (DEHP), have shown their programming effects resulting in insulin resistance in adult progeny [79,80].

Additionally, maternal substance abuse is also involved in the development of offspring MetS. In rodent models, gestational exposure to alcohol or nicotine can induce hypertension [81,82], insulin resistance [83,84] and obesity [84] in adult offspring.

The uses of drugs in pregnancy have also been connected with developmentally programmed hypertension in adult offspring, such as glucocorticoid [85], cyclosporine [86] or minocycline [87]. In addition to hypertension, early-life glucocorticoid exposure can also induce offspring’s insulin resistance [88,89,90].

In view of the fact that animal models are in line with the epidemiological observations revealing different maternal insults induce similar feature of MetS in offspring, perhaps various early-life environmental cues may mediate common mechanisms culminating in the developmental programming of MetS.

## 3. Gut Microbiota and MetS of Developmental Origins

Although the exact mechanisms underlying MetS of developmental origins have not yet been completely understood, animal studies have provided insights on potential mechanisms, including oxidative stress [90,91], dysfunctional nutrient-sensing signals [91], epigenetic regulation [92], aberrant activation of the renin–angiotensin-aldosterone system (RAAS) [92,93] and gut microbiota dysbiosis [94,95]. Notably, gut microbiota dysbiosis is interrelated to most of the above-mentioned mechanisms.

Although growing evidence supports the pathogenic interconnection between the dysbiotic gut microbiota and MetS [90,91,92], there is paucity of data about the impact of early-life disturbance of gut microbiota on offspring MetS in later life. Hence, this section primarily document evidence addressing the influence of gut microbiota on various components of MetS, with an emphasis on animal models.

### 3.1. Early-Life Gut Microbiome

Microbiota is usually defined as all the microorganisms living in a given environment, while the microbiome is a term used to describe the collection of genomes from all microorganisms in a specific environment. Though microbes will colonize the neonatal gut soon after birth [93], microbial colonization keep evolving and modulate in species abundance to attain an adult-like structure at the age of 2–3 years [94]. An important underlying contributor of offspring gut microbial structure and composition if mother microbiome [95]. Importantly, many factors can impact offspring gut microbiome, such as maternal conditions, gestational age, model of delivery, feeding type, antibiotic exposure and ecological factors [95]. Several above-mentioned risk factors connected with MetS of developmental origins have also been associated with disturbed gut microbiota, including maternal malnutrition [96], maternal obesity [97], gestational diabetes [98], LBW [99] and prematurity [100]. In addition, the establishment of the microbiome has a strong connection with developing immune system, which closely ties inflammation to MetS [61]. All of these studies indicate that adverse environmental insults induce microbial alterations may contribute to the development of MetS later in life.

### 3.2. Dysbiotic Gut Microbiota and MetS of Developmental Origins

Disruption of gut microbiota participates in the development of several MetS phenotypes, such as hypertension [101], obesity [102], dyslipidemia [103] and insulin resistance [104]. Additionally, decreased gut microbial diversity and richness are linked to a high risk for CVD [11,105].

The absence of microbiota in germ-free rats gave rise to relative hypotension in comparison with their conventional counterparts, indicating a vital role of gut microbiota in the regulation of BP [106]. In several hypertensive rat models [107,108,109], gut microbiome is disturbed and significantly different from the microbiota of normotensive control rats.

Gut microbiota metabolites are also involved in MetS of developmental origins. Short chain fatty acids (SCFAs) are products of fermentation of polysaccharides by gut microbiota. SCFAs are commonly accepted to control BP through activating their SCFA receptors [110]. Moreover, SCFAs modulate glucose homeostasis, appetite regulation and obesity [111]. Another example is trimethylamine-N-oxide (TMAO), a molecule generated from choline and carnitine via gut microbial metabolism [112]. TMAO is transformed from trimethylamine (TMA) by flavin-containing monooxygenase (FMO). High TMAO and TMO link to CVD mortality [113,114]. TMAO also contributes to MetS and associated disorders, such as type II diabetes, insulin resistance, non-alcoholic fatty liver disease and chronic kidney disease [112].

A maternal high-fat diet has been generally used to evaluate the mechanisms of MetS of developmental origins, as this model induces all features of MetS in adult rat offspring [7]. Prior work revealed that maternal high-fat diet caused offspring hypertension coincided with alterations of gut microbiota composition, reduced fecal SCFA level, dysregulated SCFA receptor expression and increased TMA levels and decreases of TMAO-to-TMA ratio in adult rat offspring [101,115]. Moreover, several indole derivatives generated from tryptophan by microbial metabolism may participate in MetS pathogenesis via activating AhR signaling [116,117]. Dysbiotic gut microbiota can mediate AhR signaling resulting in metabolic impairments, particularly liver steatosis and glucose dysmetabolism [118].

**Figure 1:**
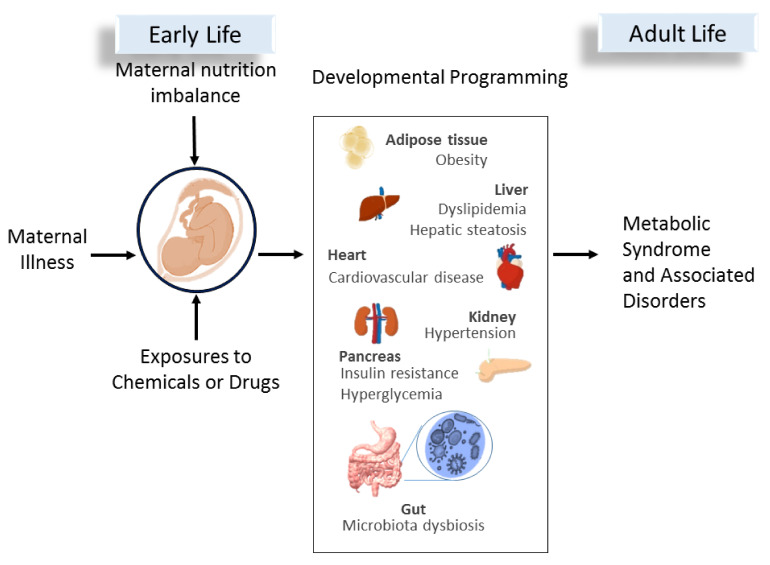
A schematic depiction delineating early-life environmental cues that may cause the developmental programming in different organ systems leading to MetS and associated disorders in adult life.

Moreover, other gut-microbiota metabolites such as lipopolysaccharide (LPS), long-chain fatty acid and bile acids (BAs) have also been linked to the MetS traits. LPS could induce low-grade inflammation, which features obesity and insulin resistance [119]. Another report showed that long chain fatty acids derived by gut microbes could be one of the mechanisms implicated in the anti-inflammatory properties of probiotics [120]. Gut microbiota-derived long chain fatty acids also play a vital role in host metabolism and adipocyte thermogenesis, which mediate anti-obesity effects [121,122,123]. Additionally, gut microbiota can convert primary BAs to secondary BAs to balance the BA pool and regulate lipid metabolism. High-fat diet-induced hyperlipidemia is related to impaired BA metabolism [124]. Figure 1 is a graphic illustration of environmental cues in early life mediate gut microbiota dysbiosis and program different organ systems, leading to MetS of developmental origins later in life.

### 3.3. Common Mechanisms behind MetS Linking to Gut Microbiota

The pathogenic interconnections between the gut microbiota and certain mechanisms are implicated in MetS of developmental origins. These core mechanisms include aberrant activation of the RAAS, oxidative stress, NO deficiency and dysregulated nutrient sensing signals [4,5,6,7,9,10,11,12].

First, activation of the RAAS can induce various phenotypes of MetS, including insulin resistance, hypertension, obesity and hyperglycemia [125]. The most common studied phenotype of MetS connected with the RAAS is hypertension [126]. There is a bidirectional interaction between the gut microbiota and RAAS; gut microbiota-derived metabolites can moderate the gut RAAS, whereas alterations in RAAS shift microbial structure and composition [127]. Angiotensin-converting enzyme 2 (ACE2), a homologue of ACE, converts angiotensin (ANG) II to ANG-(1–7) that adversely regulates the RAAS [128]. Previous studies showed that ACE2 not only can modulate gut microbiota but also alleviate hypertension and cardiovascular dysfunction in adult rat offspring [126,129,130]. Importantly, ACE2 activation has shown benefits of anti-obesity and improvement of metabolic parameters, such as blood glucose and lipids [131,132,133].

Second, data from several animal models supports a connection between gut microbiota dysbiosis and oxidative stress in the pathogenesis of developmental programming [57,134,135,136]. Enteric microbial communities govern redox signaling to maintain host–microbiota homeostasis [137]. Conversely, an imbalanced redox state induces gut microbiota dysbiosis. A maternal high-fructose diet has been reported to motivate many characteristics of MetS in adult offspring [134]. In particular, oxidative stress is close lined to dyslipidemia [138], insulin resistance [139] and hypertension [140]. Conversely, early interventions targeting gut microbiota have shown beneficial effects against oxidative stress as well as many adverse offspring outcomes in the maternal high-fructose diet rat model [141,142]. Likewise, perinatal gut microbiota-targeted therapy using resveratrol prevented the rise of BP programmed by maternal CKD in adult offspring, which coincided with altering the gut microbiota and reducing oxidative stress concurrently [132].

Third, increasing evidence suggests that NO deficiency is involved in developmental programming and has a key role in the pathogeneses of MetS [143,144]. NO deficiency can be induced by enhancing asymmetric dimethylarginine (ADMA) production, a NO synthase inhibitor [145]. A high ADMA level is connected with MetS-related disorders, such as hypertension, hypercholesterolemia, diabetes mellitus, obesity and coronary artery disease [145]. Decreased NO bioavailability and increased plasma ADMA levels have been shown to participate in several models of developmental programming [144]. As dietary nitrate (a precursor of NO) and NO metabolism can be mediated by microbiome [146], NO deficiency may work with the dysbiotic gut microbiota under the developmental programming of MetS. Resveratrol is a commonly used nutritional supplement with prebiotics and antioxidant properties [147,148]. The positive actions of resveratrol against developmental programming of hypertension are likely related to its ability to restore the ADMA/NO pathway as well alter gut microbiota in a maternal CKD model [149] and a maternal NO deficiency model [150].

Last, nutrient-sensing signals govern metabolic homeostasis in response to maternal insults during fetal development [151,152]. Hence, dysregulated nutrient-sensing signals have a crucial influence in the pathogenesis of MetS of developmental origins [7]. Gut microbiota-diet interactions interfere in nutrient-sensing signals from the gut to the brain, where the information is processed to govern whole-body metabolic and energy homeostasis [153]. It has long been known that cyclic adenosine monophosphate (AMP)-activated protein kinase (AMPK) is a key nutrient-sensing signal. Dysfunctional AMPK signal is related to developmental programming of hypertension, while AMPK activation in early life could prevent offspring hypertension [154]. Additionally, resveratrol, an AMPK activator, can regulate nutrient-sensing signals to increase expression of PPARs target genes and thereby reverse MetS-related programmed processes [9,147].

With regard to the multifaceted role of gut microbiota in human health, other possible pathways might be interconnected and all work together to program MetS, for example, hydrogen sulfide signaling [155] or nuclear factor erythroid 2-related factor 2 (NRF2) [156]. Although the exact mechanism behind MetS of developmental origins remains inconclusive, animal studies provide a possibility regarding gut microbiota as a possible reprogramming target.

## 4. Reprogramming Strategy: Probiotics, Prebiotics and Postbiotics

The DOHaD theory generates opportunities to stop or delay the programming process by an early reprogramming strategy aiming to prevent adult disease later in life [15]. With a deeper understanding on MetS programming, the development of mechanism-targeted strategies provides potential for reprogramming. Emerging evidence from animal studies in DOHaD research supports that gut microbiota-targeted therapy might act as a reprogramming strategy to avert adult disease of developmental origins [11].

### 4.1. Gut-Microbiota Targeted Therapy

Several gut microbiota-targeted therapies have proven to manipulate the gut microbiome in various disorders. Probiotics and prebiotics are the most frequently used gut microbiota-targeted options in clinical work [16,17,18]. Probiotics refers to live microorganisms that, when administered in adequate amounts, confer a health benefit on the host [157]. The international scientific association of prebiotics and probiotics (ISAPP) defined prebiotics as substrates that are selectively utilized by host microorganisms conferring a health benefit [158]. Synbiotics, a probiotic-prebiotic combination, also confers a health benefit [16].

Postbiotics and parabiotics and the emerging concepts in the functional foods field, which have shown to promote health, too [159]. The postbiotics are the complex mixture of metabolic bioproducts generated by probiotics in cell-free supernatants such as vitamins, enzymes, organic acids, secreted proteins, amino acids, SCFAs, peptides and secreted biosurfactants. While the parabiotics are the inactivated microbial cells of probiotics or crude cell extracts [159]. Another way to modify the gut microbiome is by transplanting fecal matter. Emerging evidence suggests efficacy of fecal microbiota transplant (FMT) for the therapy of obesity associated disorders [160].

Here, we illustrate Table 1 that summarizes studies reporting microbiota-targeted reprogramming interventions in animal models for studying MetS of developmental origins, restricting those therapeutic duration is starting before birth to cover the periods of organogenesis [57,101,141,142,149,150,161,162,163,164,165,166,167,168,169,170,171,172,173,174,175,176,177,178].

The most widely used species are rats. A number of MetS programming models have been used to examine gut microbiota-targeted interventions, such as maternal high-fructose diet [141,142], perinatal high-fat diet [57,101,167,169], maternal high-fat/sucrose diet [162], maternal high-fat diet [161,163,171,176], maternal ADMA and TMAO exposure [164], perinatal TCDD exposure [165,177], maternal adenine-induced CKD [149], maternal protein restriction [165], maternal L-NAME and high-fat diet exposure [150], maternal bisphenol A exposure and high-fat diet [162], maternal hypertension [170], maternal bisphenol A exposure [172,173], maternal minocycline exposure [174], maternal tryptophan-free diet [175] and combined maternal high-fructose diet and TCDD exposure. [173].

**Figure 2:**
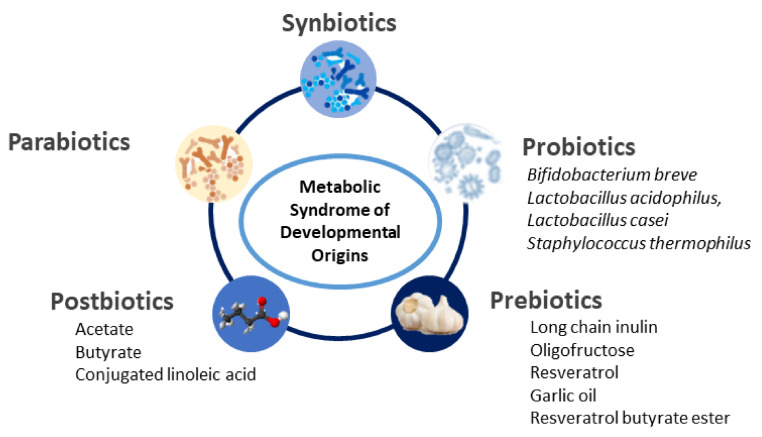
A summary of the currently available reprogramming interventions for metabolic syndrome of developmental origins.

Reported gut microbiota-targeted strategies include probiotics, prebiotics and postbiotics. A schematic summary of gut microbiota-targeted reprogramming interventions for MetS of developmental origins is illustrated in Figure 2.

In view of the fact that the difficulties in developing animal models exhibiting all characteristics of MetS, gut microbiota-targeted interventions into developmental programming of MetS have been evaluated for their protective effects against some but not all characteristics of MetS. Table 1 illustrates maternal high-fat diet induces almost all characteristics of MetS in adult offspring at 3–24 weeks of age, such as obesity [54,57,60,62,63], hypertension [57,101,141,142,149,150,161,162,163,164,165,166,167,168,169,170,171,172,173,174,175,176,177,178], dyslipidemia [60,62,63], hepatic steatosis [162,169,173], insulin resistance [161,162,166] and CVD [176]. Hypertension is the most commonly studied phenotype of MetS.

### 4.2. Probiotics

The major probiotics consist of one or mor strains coming from the genera *Lactobacillus* spp. and *Bifidobacterium* spp. [16,17,18]. A recent systematic review reported that probiotics supplementation in patients with MetS improved obesity, hypertension, glucose metabolism and dyslipidemia [179]. In spite of probiotics demonstrating benefits in MetS [179], there was scant evidence with respect to their impact on MetS of developmental origins. Using the perinatal high-fat diet [101] or high-fructose diet [141] rat model, the use of *Lactobacillus casei* during gestation and lactation periods has shown to benefits on hypertension in adult progeny. Another study showed that maternal multi-strain probiotics supplementation (*Bifidobacterium breve*, *Lactobacillus acidophilus*, *Lactobacillus casei* and *Staphylococcus thermophilus*) improved glucose and insulin levels in female mice offspring programmed by maternal high-fat diets [161].

### 4.3. Prebiotics

Dietary fibers, such as inulin or oligosaccharides, are the best-known prebiotics [18]. Inulin supplementation during gestation and lactation has been reported to protect adult rat offspring against hypertension induced by maternal high-fructose or high-fat diet [101,141]. Another study tested the maternal high-fat/sucrose diet model and revealed that modulation of gut microbiota by oligofructose can avert insulin sensitivity, hepatic steatosis and glucose tolerance in adult progeny [162].

In addition to fibers, a large proportion of foods remains unabsorbed and are metabolized by the gut microbiota. These dietary contents, such as garlic and polyphenols, have shown prebiotic-like effects [180,181]. Although there are many prebiotic foods, Table 1 shows that only garlic and resveratrol have shown benefits on protection of MetS in adult offspring. The protective effects of maternal garlic oil treatment against high-fat diet-induced offspring hypertension accompanying by enhanced α-diversity; increased plasma levels of acetate, butyrate and propionate; and augmented abundance of beneficial microbes *Bifidobacterium* and *Lactobacillus* [57].

### 4.4. Resveratrol

Polyphenols are the greatest group of phytochemicals. The use of polyphenols as a reprogramming intervention has been examined in animal models of developmental hypertension [182]. One of the most extensively studied groups of polyphenols is resveratrol [182]. Importantly, resveratrol has been proposed as a reprogramming strategy for preventing MetS programming [183].

Table 1 shows that the use of resveratrol before birth has beneficial effects against adverse offspring outcomes, including obesity [163,166,169,171], hypertension [149,164,165,167,168,170], insulin resistance [166], hepatic steatosis [169] and hyperlipidemia [169,171] in various MetS programming models.

Resveratrol prevented maternal FCDD exposure-induced offspring hypertension was related to alterations of the gut microbiota by enhancing microbes that can inhibit T helper 17 cell (TH17) responses and diminishing the *Firmicutes* to *Bacteroidetes* (F/B) ratio [165]. Additionally, perinatal resveratrol therapy prevented adult offspring from maternal CKD-induced hypertension, which was associated with restoration of microbial richness and diversity and an increase in beneficial microbes, *Bifidobacterium* and *Lactobacillus* [149].

However, the low bioavailability of resveratrol restricts its clinical translation [184]. On this matter, resveratrol was esterified to resveratrol butyrate esters (RBE), to enhance the efficacy and facilitate broad applications [185]. Our recent study demonstrated that low-dose RBE (30 mg/L) is able to protect against maternal bisphenol A exposure-induced obesity and hyperlipidemia [172] in female progeny and hepatic steatosis in male progeny [173] in a sex-specific manner.

Although some prebiotics have shown benefits in offspring MetS-related disorders, much remains unclear regarding the interplay between gut microbiota and prebiotics and the impact of prebiotic foods as a reprogramming strategy for MetS of developmental origins.

### 4.5. Postbiotics

SCFAs are the main microbial metabolites and can serve as postbiotics. One previous study reported that acetate supplementation during pregnancy and lactation periods was able to prevent offspring against hypertension programmed by maternal high-fructose diet [142] or maternal minocycline exposure [174]. Another study examined the maternal tryptophan-free diet model and found that modulation of gut microbiota by maternal butyrate supplementation can protect the development of hypertension in adult progeny [175]. Conjugated linoleic acid is a gut microbiota-derived metabolite from dietary polyunsaturated fatty acids. As a postbiotic, maternal conjugated linoleic acid supplementation reversed maternal high-fat diet-induced offspring hypertension [176]. Since postbiotics cover a wide range of bioactive compounds produced by microorganisms, the reprogramming effects of other postbiotics on various characteristics of MetS are awaiting further clarification.

### 4.6. Others

Another way to manipulate the gut microbiome is to regulate microbial metabolites. For example, microbe-dependent TMA and TMAO formation can be inhibited by a structural analog of choline, 3,3-dimethyl-1-butanol (DMB) [186]. In a maternal high-fructose diet model, maternal DMB treatment protected adult rat offspring against hypertension, which was coincided with the reduction of TMA and TMAO levels [142]. Similarly, the use of DMB in pregnancy and lactation as a reprogramming intervention to prevent offspring hypertension has been proven in a maternal TCDD exposure model [177] and a combined TCDD and high-fructose exposure model [178].

## 5. Translating Animal Models to Clinical Practice

Animal studies support that early use of certain probiotics, prebiotics or postbiotics may prevent MetS of developmental origins, while this growing body of evidence awaits translating into clinical practice.

In clinical work, the most generally used treatment options to manipulate gut microbiota are probiotics and prebiotics. When discussing the therapeutic benefits of probiotics and prebiotics in clinical practice, special consideration should be paid to their safety. So far, probiotic or prebiotics supplementation during pregnancy are limited in human studies [187]. Limited information that currently exists suggests that probiotic supplementation for pregnant women is mostly safe and may have a beneficial role in gestational diabetes [188], preeclampsia [189], vaginal infections [190], obesity, [191] and spontaneous preterm delivery [192]. However, little reliable information is available about the uses of various prebiotic-like components or prebiotic-rich food, either individually or in combination, in pregnant women [193]. More importantly, currently no information exists regarding their effectiveness in protecting adult disease or long-term safety in offspring.

In the context of safety, postbiotics and parabiotics are safer as compared to probiotics. Unlike definitions were provided by the ISAPP and the Food and Agriculture Organization of the United Nations-WHO (FAO-WHO) for probiotics and prebiotics, currently there remains a lack of a clear definition for postbiotics and parabiotics. Considering the complex nature of postbiotics and parabiotics, there is urgent need to define both terms clearly from a regulatory perspective.

Currently, no information regarding the impact of probiotic or prebiotics supplementation during pregnancy on long-term offspring outcome related to MetS is available in human studies. As review elsewhere [194,195], prior studies investigating the impact of maternal probiotics or prebiotics supplementation on offspring outcome have mainly focused on allergic or metabolic diseases as main outcomes. Nevertheless, the offspring outcome in almost all studies are only determined in neonatal or infantile period.

Recently, several clinical trials were completed in overweight women and women diagnosed with gestational diabetes [191,196,197,198,199]. Mid- or late-pregnancy supplementation with several mixtures of *Lactobacillus*, *Bifidobacterium* and *Streptococcus* species had no impact on anthropometric measures at birth. Nevertheless, their long-term effects on metabolic outcomes are still unknown. Although there are more than 10 ongoing trials working on probiotic or prebiotics supplementation during pregnancy [200], none of them focus primarily on offspring outcome related to MetS and associated disorders. To briefly sum up, maternal prebiotic and probiotic interventions in animals show promising results; however, transferability to the human trial is yet to be confirmed. Accordingly, future work in large prospective trials is required to better identify probiotic species and improve formulation of prebiotics for MetS of developmental origins.

## 6. Conclusions and Future Perspectives

Previous research has indicated the impact of the gut microbiota in MetS and associated disorders. This review sought to highlight disturbance of gut microbiota during fetal development linking to MetS in later life. Our review also, reflecting current knowledge, opens a new window for preventing MetS of developmental origins via gut microbiota-targeted reprogramming strategies.

No matter recent advances in building appropriate animal models for studying developmental programming of MetS, only few models exhibit the full characteristics of MetS. Even though several gut microbiota-targeted interventions have brought about a significant progress in certain components of MetS in one model, attention should be given to clarify whether their reprogramming effects are also advantageous for other MetS phenotypes. After all this tremendous growth in gut microbiota-targeted interventions and deeper understanding of MetS programming, we expect that microbiota-based reprogramming therapies will be employed in clinics to reduce the global burden of MetS and associated disorders.

## Figures and Tables

**Table 1:** Summary of animal models reporting gut microbiota-targeted therapies for MetS of developmental origins.

Gut Microbiota-Targeted Therapies	Animal Models	Species/Gender	Age at Evaluation	Reprogramming Effects	Ref.
Probiotics					
Daily oral gavage of *Lactobacillus casei* during gestation and lactation	Maternal high-fructose diet	SD rat/M	12 weeks	Prevented hypertension	[141]
Daily oral gavage of *Lactobacillus casei* during gestation and lactation	Perinatal high-fat diet	SD rat/M	16 weeks	Prevented hypertension	[101]
Daily oral gavage of multi-strain probiotics (*Bifidobacterium breve*, *Lactobacillus acidophilus*, *Lactobacillus casei* and *Staphylococcus thermophilus*) during gestation and lactation	Maternal high-fat diet	C57BL/6 J mice/F	20 weeks	Improved glucose and insulin levels	[161]
Prebiotics					
5% *w*/*w* long chain inulin during gestation and lactation	Maternal high-fructose diet	SD rat/M	12 weeks	Prevented hypertension	[141]
5% *w*/*w* long chain inulin during gestation and lactation	Perinatal high-fat diet	SD rat/M	16 weeks	Prevented hypertension	[101]
10% *w*/*w* oligofructose during gestation and lactation	Maternal high-fat/sucrose diet	SD rat/M	24 weeks	Improved glucose tolerance, insulin sensitivity and hepatic steatosis	[162]
Daily oral gavage of garlic oil (100 mg/kg/day) during gestation and lactation	Perinatal high-fat diet	SD rat/M	16 weeks	Prevented hypertension	[57]
Resveratrol (50 mg/L) in drinking water during gestation and lactation	Maternal high-fat diet	Wistar rat/M and F	3 weeks	Improved obesity	[163]
Resveratrol (50 mg/L) in drinking water during gestation and lactation	Maternal ADMA and TMAO exposure	SD rat/M	12 weeks	Prevented hypertension	[164]
Resveratrol (50 mg/L) in drinking water during gestation and lactation	Perinatal TCDD exposure	SD rat/M	12 weeks	Prevented hypertension	[165]
Resveratrol (50 mg/L) in drinking water during gestation and lactation	Maternal adenine-induced CKD	SD rat/M	12 weeks	Prevented hypertension	[149]
Daily oral gavage of resveratrol (20 mg/kg/day) during gestation	Maternal protein restriction	Wistar rat/M and F	110 days	Improved obesity and insulin resistance	[166]
Resveratrol (50 mg/L) in drinking water during gestation and lactation	Maternal L-NAME administration and high-fat diet	SD rat/M	16 weeks	Prevented hypertension	[150]
Resveratrol (50 mg/L) in drinking water during gestation and lactation	Maternal and post-weaning high-fat diet	SD rat/M	16 weeks	Prevented hypertension	[167]
Resveratrol (50 mg/L) in drinking water during gestation and lactation	Maternal bisphenol A exposure and high-fat diet	SD rat/M	16 weeks	Prevented hypertension	[168]
Resveratrol (50 mg/L) in drinking water during gestation and lactation	Maternal and post-weaning high-fat diet	SD rat/M	16 weeks	Improved obesity, hyperlipidemia and hepatic steatosis	[169]
Resveratrol (4 g/kg of diet) during gestation and lactation	Maternal hypertension	SHR/M and F	20 weeks	Prevented hypertension	[170]
Resveratrol (0.2% *w*/*w*) during gestation and lactation	Maternal high-fat diet	C57BL/6 J mice/M	14 weeks	Improved obesity and hyperlipidemia	[171]
Daily oral gavage of resveratrol butyrate ester (30 or 50 mg/kg/day) during gestation and lactation	Maternal bisphenol A exposure	SD rat/F	50 days	Improved obesity and hyperlipidemia	[172]
Daily oral gavage of resveratrol butyrate ester (30 mg/kg/day) during gestation and lactation	Maternal bisphenol A exposure	SD rat/M	50 days	Improved hepatic steatosis	[173]
Postbiotics					
Magnesium acetate (200 mmol/L) in drinking water during gestation and lactation	Maternal high-fructose diet	SD rat/M	12 weeks	Prevented hypertension	[142]
Magnesium acetate (200 mmol/L) in drinking water during gestation and lactation	Maternal minocycline exposure	SD rat/M	12 weeks	Prevented hypertension	[174]
Sodium butyrate (400 mg/kg/day) in drinking water during gestation and lactation	Maternal tryptophan-free diet	SD rat/M	12 weeks	Prevented hypertension	[175]
1% conjugated linoleic acid in chow during gestation and lactation	Maternal high-fat diet	SD rat/M	150 days	Improved cardiometabolic dysfunction	[176]
Others					
1% DMB in drinking water during gestation and lactation	Maternal high-fructose diet	SD rat/M	12 weeks	Prevented hypertension	[142]
1% DMB in drinking water during gestation and lactation	Perinatal TCDD exposure	SD rat/M	12 weeks	Prevented hypertension	[177]
1% DMB in drinking water during gestation and lactation	Maternal high-fructose diet and TCDD exposure	SD rat/M	12 weeks	Prevented hypertension	[178]

Studies tabulated based on types of intervention, animal models and age at evaluation. CKD = chronic kidney disease; TCDD = 2,3,7,8-tetrachlorodibenzo-p-dioxin; ADMA = asymmetric dimethylarginine; TMAO = trimethylamine-N-oxide; SD = Sprague-Dawley rat; DMB = 3,3-maternal dimethyl-1-butanol.

## Data Availability

All data are contained within the article.
